# “Don’t think of a soda”: Contradictory public health messaging from a content analysis of Twitter posts about sugar-sweetened beverage taxes in California from 2015 to 2018

**DOI:** 10.3389/fpubh.2024.1390253

**Published:** 2024-07-09

**Authors:** Kim Garcia, Pamela Mejia, Sarah Perez-Sanz, Lori Dorfman, Kristine Madsen, Dean Schillinger

**Affiliations:** ^1^Berkeley Media Studies Group, Berkeley, CA, United States; ^2^School of Public Health, University of California, Berkeley, Berkeley, CA, United States; ^3^School of Medicine, University of California, Berkeley, San Francisco, CA, United States

**Keywords:** nutrition, health policy, SSB taxes, advocacy, communications

## Abstract

To show how sugar-sweetened beverage (SSB) taxes were framed in posts on Twitter (now known as X) through text and images, we conducted a content analysis on a sample of Tweets from California users posted between January 1, 2015 and December 31, 2018 about SSB taxes in Berkeley, San Francisco, Oakland, and/or Albany, California. We evaluated posts for information sources, arguments for or against SSB tax policies, and images used. We found that posts presented a mix of messages through text and images. The majority of posts (64%) included arguments supporting SSB taxes, 28% presented a neutral position (e.g., factual information) or a mix of both pro-and anti-tax arguments, and 8% opposed. One-third of posts included an image, almost half of which appeared to be stock photos from SSB advertisements: many of these were shared by medical and public health users. Some tax supporters also reposted messages and images from opposition campaigns and added their own criticisms. By reposting opponents’ anti-tax messages and images of SSBs, tax supporters may have inadvertently promoted SSBs, reinforced opposition to SSB taxes, and normalized SSBs. While advocates effectively shared pro-tax arguments, they should also ensure that accompanying images reflect the solutions they seek, not just the problem they are trying to combat.

## Introduction

Public health advocates have developed strategies to reduce the harms of sugar-sweetened beverages (SSBs), including SSB taxes, and the evidence is clear: SSB taxes benefit communities. Studies show that SSB taxes reduce sales and consumption of SSBs (1–3) and generate revenue for public health initiatives (4, 5). The implementation of over 100 excise taxes on SSBs around the world shows the increasing popularity and success of these public health policies (6).

However, advocates of SSB taxes face intense opposition from the beverage industry, which borrows strategies from the “corporate playbook” of industries like tobacco (7). One industry strategy is influencing how health issues are framed in public debates. “Framing” refers to how an issue is portrayed and understood; in written and visual media, the selection or exclusion of words, arguments, or images can prompt values, beliefs, and feelings (8). For example, SSB industry actors use arguments highlighting personal responsibility and freedom to deflect attention from calls for industry accountability (7). Further, despite industry marketing practices that disproportionately target low-income communities and communities of color (9), some industry campaigns also align themselves with those communities by depicting images of working class people and local, small business owners, whom they argue would be “unfairly harmed” by SSB taxes (10).

Recent studies demonstrate how advocates can use framing and messaging to advance support for SSB taxes in traditional news media (11). However, less is known about how SSB taxes are framed in social media – an increasingly important source of information for voters and the general public (12). We built upon research examining trends in SSB tax sentiments of posts on Twitter (13) (sold and re-branded as ‘X’ as of July 2023), by evaluating images as well as text used in Twitter posts. Images play an important role in health communications because pictures – when closely aligned with text – can increase memorability, while also benefiting comprehension, especially among lower literacy or non-English-speaking groups (14). Some research demonstrates that images can enhance the engagement and sharing (or diffusion) of social media posts (15). For example, recent studies analyzing Twitter posts about COVID-19 vaccines found that posts with images were twice as likely to be shared than their text-only counterparts (16).

Our aim was to evaluate messaging about SSB tax policies in Twitter posts and consider how the text and images used may have strengthened – or undermined – arguments for SSB taxes. We chose to evaluate Twitter posts because they appear on a publicly accessible social media platform offering content that can be immediately seen by anyone. In addition, during the period of our analysis, Twitter allowed free and unlimited access to its data for research purposes. Twitter is a unique and powerful communication platform because it creates opportunities for users to translate complex scientific studies into plain, more accessible language because of character limits that were in effect during the period of our analysis. On the other hand, Twitter, like other social media platforms, can serve as a major source of misinformation and disinformation (17). Therefore, we were also interested in seeing how Twitter text and associated images may have generated content with potential unintended consequences for advocates using the platform to advance support for SSB tax policies.

## Methods

We evaluated Twitter posts or “Tweets” about campaigns in four California cities (Berkeley, San Francisco, Oakland, and Albany) where residents voted on SSB taxes between 2014 and 2018. To learn how advocates characterized SSB taxes in Twitter posts, we used the social media software Keyhole to collect posts published between January 1, 2015 and December 31, 2018 that referenced campaigns in one of the four cities. We could not collect posts from 2014 as Twitter prohibited data collection from before January 1, 2015.

We collected posts that included at least one of the following terms or hashtags: “soda tax,” “drink tax,” “beverage tax,” “sin tax,” “SSB tax,” #SodaTax, #SSBTax, or #SinTax. We also added location-related terms (i.e., Berkeley, San Francisco, Oakland, Albany) and variations (e.g., “SF” or “Bay Area”) because Keyhole’s capacity to narrow a search for posts by location was not reliable. Qualifying posts were geotagged within the United States; however, due to the initial high volume of search results, we further limited our sample to those posted by users who self-identified as being located in California in their profile or “bio.” We randomly selected a 15% sample (n = 715 posts) for content analysis.

We adapted a codebook from prior analyses of news articles about SSB taxes (18) incorporating social media elements, such as whether the post duplicated content from other users (such as a “re-tweet” or “quote tweet”). First, we assessed if the post met our *relevance* criteria: we included posts about SSB tax policies in Berkeley, San Francisco, Oakland, or Albany. We excluded posts about SSB taxes in other cities, unrelated propositions or taxes, or if they did not have enough context to understand the post. We evaluated each relevant post for *sources* by identifying the type of user account that published the post, as well as credits to other authors quoted or “re-tweeted” in the post. We reviewed how SSB taxes were framed through the types of *arguments* that appeared (i.e., SSB taxes work/do not work, SSB taxes are necessary/unnecessary, SSB taxes are helpful/harmful) and the types of *images* depicted (e.g., SSBs, sugar, children, vegetables, etc.). A full list of variables for analysis in the coding instrument can be found in Table 1.

**Table 1:** Coding instrument used to evaluate Twitter posts about SSB taxes in Berkeley, San Francisco, Oakland, and Albany, from 2015 to 2018.

**Research question**	**Variable**	**Code options**
1. Is the post relevant?	1. Relevance	Relevant: Post mentions SSB taxes in California Irrelevant or cannot code: Post mentions SSB taxes outside California or does not contain enough context to understand the post
2a. Who wrote the original post? 2b. Does the post quote another source? 2c. Does the post credit another source?	2a. Source of original post 2b. Source quoted in post 2c. Source credited in post	Local government official: Alameda or San Francisco counties only; elected, non-elected, or former representatives Non-local government official: federal, state, or counties outside of Alameda or San Francisco counties; elected, non-elected, or former News outlets: Attribution to other news sources Pro-tax coalition or members: Berkeley Healthy Child Coalition, Berkeley vs. Big Soda, Vote Yes on V, Coalition for Healthy Oakland Children, Yes on O1 Campaign American Beverage Association or other beverage industry representative: Includes spokespeople, affiliated consultants, vending associations Anti-tax coalition or members: No Berkeley Beverage Tax, Californians for Food & Beverage Choice, Enough Is Enough: Do not Tax Our Groceries, No Oakland Grocery Tax - No on Measure HH, No on O1 campaign Other business representatives: Includes local or small business owners Medical and public health representative: Includes professionals, advocates, or researchers Community representative, authentic voice, or private citizens: Includes local residents, parents, youth, educational institutions, faith-based organizations, non-profit organizations Other
3. Does the post contain external links?	3. Links	Yes No
4. Does the post reference any of the following policies?	4. Policy	Measure D in Berkeley Prop V in San Francisco Measure HH in Oakland Prop O1 in Albany Statewide California SSB tax Another SSB or sugar-related policy from before 2015 (e.g., Prop E in San Francisco from 2014)
5. What is the stance of the post?	5. Stance	In favor of SSB taxes Opposed to SSB taxes Neutral or impossible to discern stance
6. Does the post contain any argument frames?	6. Arguments	Taxes work (e.g., tax will lower consumption) Taxes do not work (e.g., tax will not lower consumption) Taxes are necessary (e.g., SSBs cause health harms) Taxes are unnecessary (e.g., SSBs do not cause health harms) Taxes help (e.g., tax will improve public health) Taxes harm (e.g., tax is regressive)
7. Does the post contain any images, photos, or videos?	7. Images	Yes No
8. What types of images appear in the post?	8. Types of images	Meme, cartoon, or other generated image People – white People – not white Youth Beverage company (e.g., corporate employees or executives) Small businesses (e.g., grocery store) Health-related images – doctor’s offices, doctors, or people in white lab coats with stethoscopes SSB or sugar (e.g., SSB cans, SSB brands, piles of sugar) Non-soda or non-sugar food/drink (e.g., vegetables, milk, nuts, etc.)

We tested our coding instrument and achieved acceptable intercoder reliability levels (*Krippendorff’s alpha* > 0.8 for all variables). After removing irrelevant posts, we analyzed a total of 683 relevant posts.

## Results

### Timeline and volume

**Figure 1:**
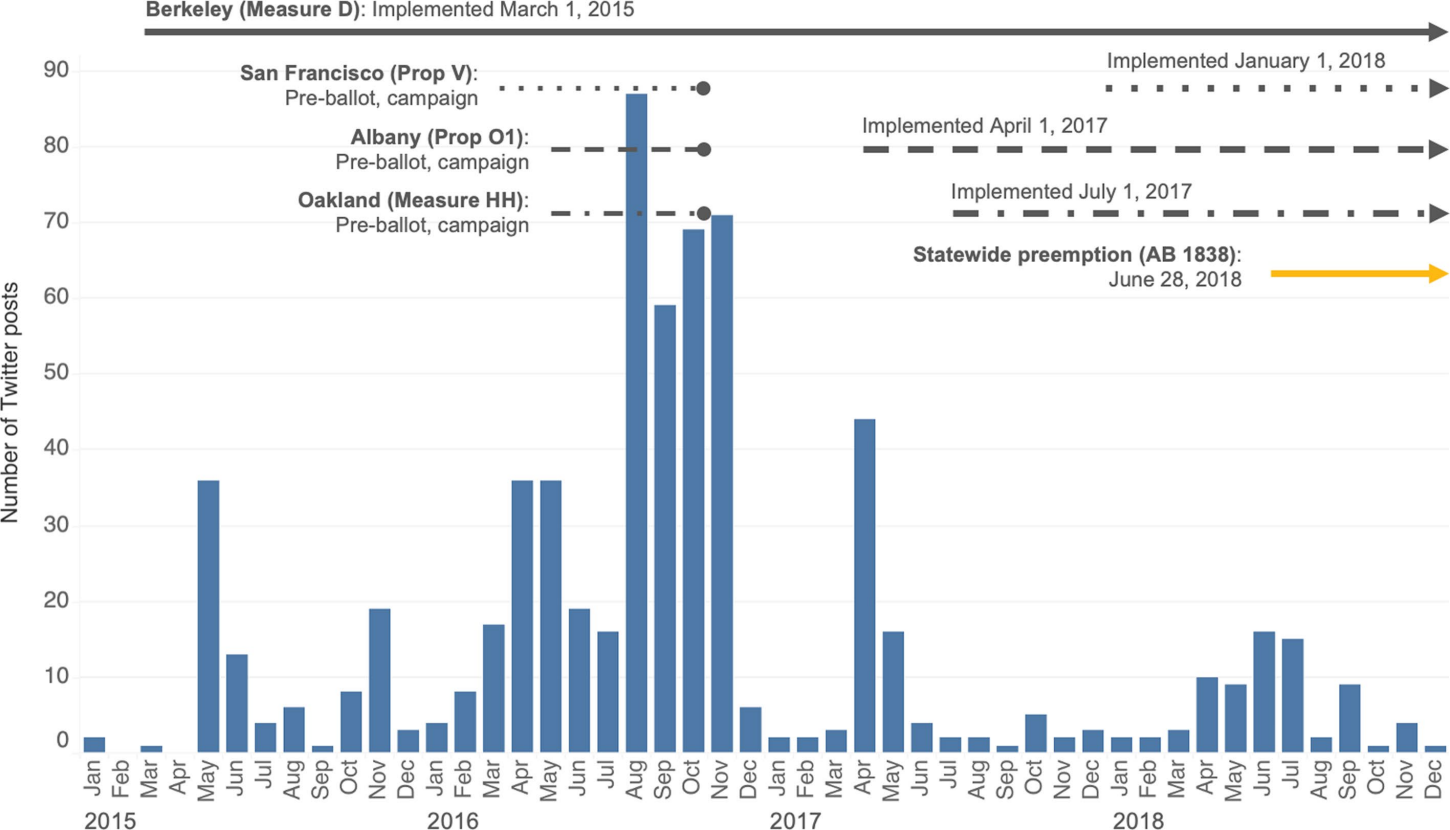
Timeline and volume of posts about SSB taxes in Berkeley, San Francisco, Oakland, and Albany, from 2015 to 2018 (*n* = 715).

The volume of posts varied over time during different phases of the policy process (see Figure 1). We observed a high volume of posts in May 2015, many of which included reports of funds raised after the implementation of the Berkeley SSB tax, and some Tweets about the proposed measure for a California statewide SSB tax, which ultimately did not pass. The highest volume of posts appeared in August 2016 during the San Francisco, Albany, and Oakland SSB tax campaigns. Posts increased in volume again in April 2017, corresponding with the implementation of the San Francisco SSB tax.

#### Sources

The majority (64%) of posts were published by or attributed to a variety of traditional print, digital, and TV news sources, such as *The San Francisco Chronicle*, *East Bay Express*, and FOX40 News, as well as from the accounts of self-identified reporters and journalists affiliated with news outlets. Medical and public health professionals were sources for about half (47%) of posts; these included individuals and organizational accounts, such as medical associations, health coalitions, and public health institutions. Community-based organization representatives (e.g., East Oakland Collective, Berkeley Youth Alternatives) and residents (i.e., self-identified local residents with no listed affiliations) were the third most frequent source; most of their content were “re-tweets” or shared content from other user accounts, without publishing their own original content. Government officials were sources in 5% of posts.

### Framing: arguments about SSB tax policies

Almost two-thirds of posts (64%) included arguments supporting SSB taxes. The majority of these posts argued that SSB taxes successfully lower consumption of SSBs, set a good precedent for other SSB tax policies, and/or effectively raise revenue for the local community. For example, one Berkeley resident posted, “In 3 years, Berkeley’s #sodatax has generated over $5 million in tax revenue for programs that improve the health of Berkeley communities” (19). Some posters argued that SSB taxes were needed because of the harmful effects of SSBs, as when a medical organization stated, “We support SF #sodatax b/c sugar sweetened beverages consumption is linked to increased risk of obesity & diabetes” (20). Others pointed out the beverage industry’s wrongdoing during campaigns. For example, an Oakland resident posted a local news article reporting on the disproportionate spending on the opposition campaign with the headline, “On the ‘grocery’ tax, the American Beverage Association’s attempt to mislead Oakland voters about the [soda] tax” (21).

Posts with arguments opposing SSB tax policies (8%) tended to describe taxes as harmful to consumers, framing taxes as infringements on personal freedom. For example, one northern California resident shared a link and quote from a blog post about the Berkeley SSB tax, which said, “…it is conceptually problematic for a third party to deem another person’s choices wrong” (22). Some posts included claims that SSB taxes do not work, as when a food and beverage industry consultant shared a report titled, “Berkeley soda tax not effective: consumers & business owners lose” (23).

### Framing: images

**Figure 2:**
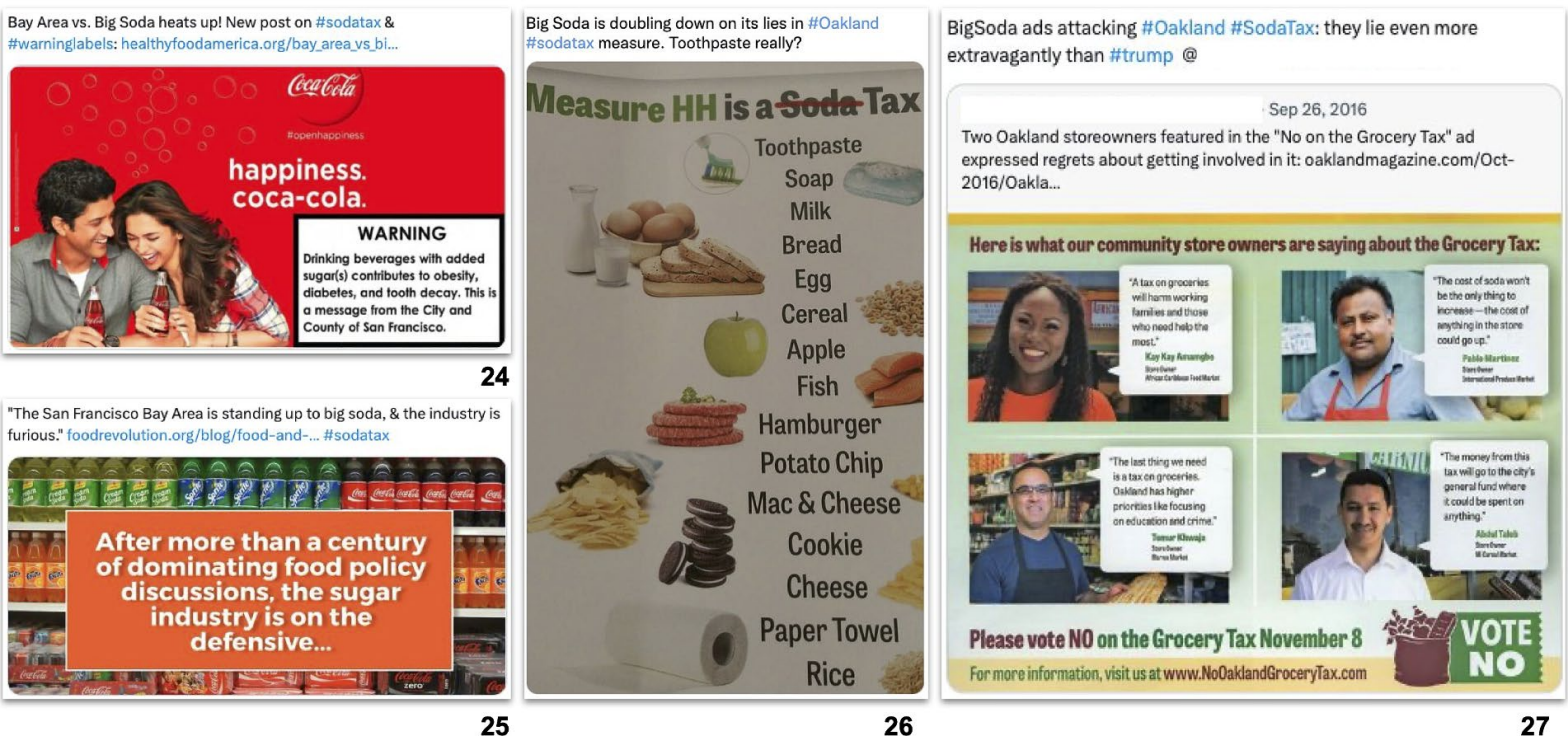
Images reinforcing opposition and industry frames in posts supporting SSB tax policies in Berkeley, San Francisco, Oakland, and Albany, from 2015 to 2018 (*n* = 715).

One-third of posts included an image. Nearly half of these images (46%) depicted SSBs themselves. News outlets frequently pictured SSBs with clear branding (51% of SSB images), such as logos and advertisements (24). Some images included multiple products and brands, such as photos of crowded shelves of SSBs in a grocery store (25). Medical and public health sources who supported SSB taxes in the text of their posts also reproduced images of SSBs, posting almost one quarter (24%) of SSB images (Figure 2).

Occasionally, posts included images from opposition campaigns that included messaging and imagery arguing against SSB taxes. Some medical and public health sources shared these images – usually with criticisms or corrections. For example, a public health lawyer criticized the beverage industry by sharing a pamphlet which misrepresented SSB taxes as “grocery taxes,” showing a range of non-SSB products that allegedly would be taxed (26). Other images were posted by tax supporters who denounced the beverage industry for their dishonest campaigns and messages, as when an Oakland resident shared an anti-tax flyer and quipped, “BigSoda ads attacking #Oakland #SodaTax: they lie even more extravagantly than #trump” (27).

**Figure 3:**
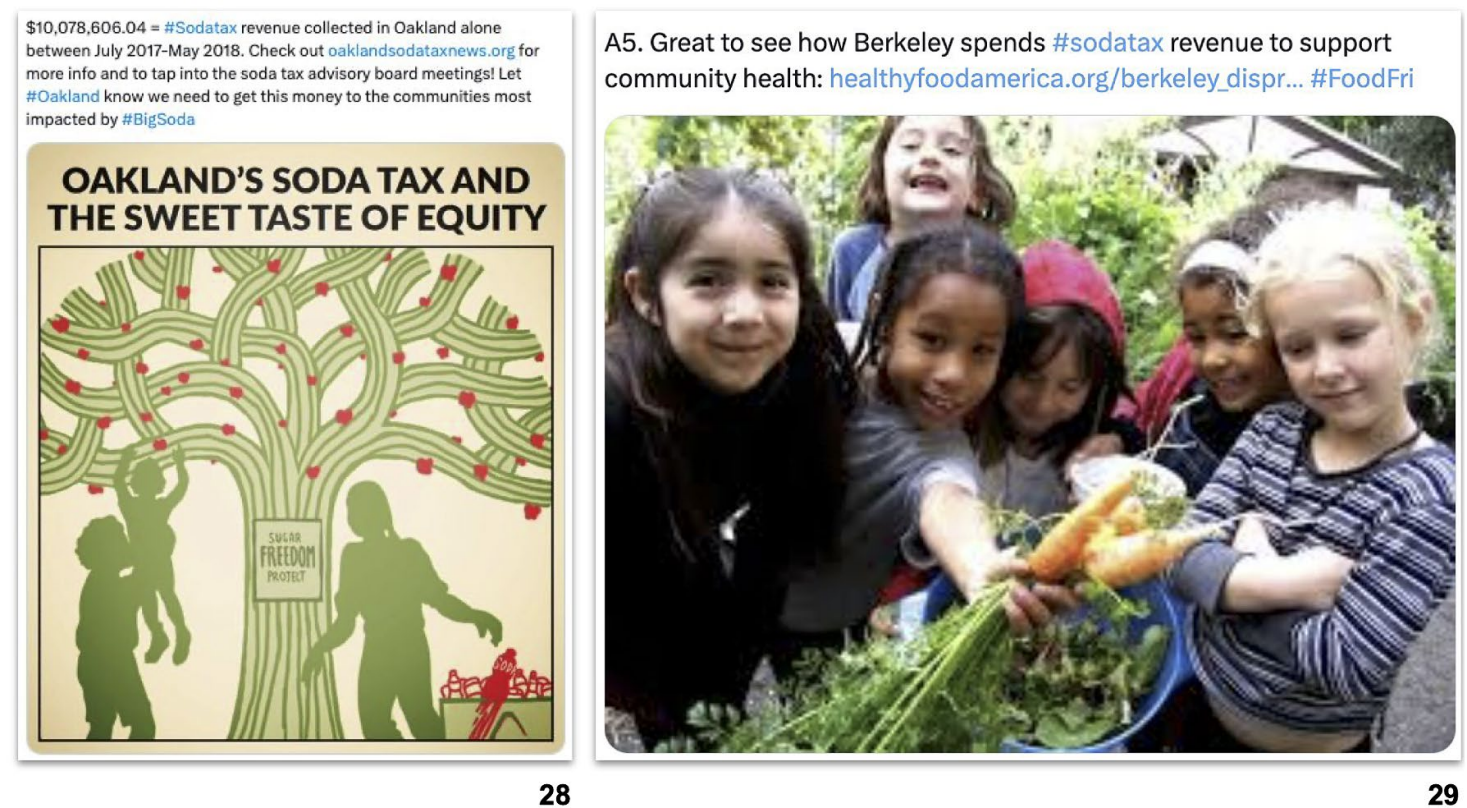
Images reinforcing advocacy and public health frames in posts supporting SSB taxes in Berkeley, San Francisco, Oakland, and Albany, from 2015 to 2018 (*n* = 715).

Not all images in support of SSB taxes reinforced SSB opponents’ frames. A few posts supporting SSB taxes posted images that focused on the outcomes of SSB taxes and communicated values like equity and community health (Figure 3). For example, one pro-tax coalition applauded the millions of dollars raised by the Oakland SSB tax within its first year and accompanied the post with an image of the city’s emblematic oak tree with fruits to represent “the sweet taste of equity” (28). Another example from a healthy food advocacy organization praised the Berkeley SSB tax and highlighted the benefits of the revenue raised with an image depicting children in a community garden holding fresh vegetables (29).

## Discussion

Our analysis showed that many Twitter posts about SSB taxes overtly argued in favor of those policies in four California cities, a pattern we also saw in print and online news coverage (30). News outlets were key sources of information, followed by medical and public health professionals, and community representatives. Many of these posts presented pro-SSB tax messages in effective ways that bolstered their case. However, some tax supporters reposted anti-tax materials with misinformation as part of attempts to expose or critique beverage industry tactics, or reproduced appealing images of SSBs such as those used to advertise the product. By reposting images of anti-tax materials and SSBs, advocates may have undermined the intent of their messaging.

These findings are important from an advocacy perspective because reproducing images of opposition campaign materials may create barriers to illustrating why SSB taxes matter for community health. Work by cognitive linguist George Lakoff (31) illustrates a mechanism by which tax supporters on Twitter may have unconsciously distracted from their own messages when they presented images of anti-tax materials including opposition arguments. We refer to these mechanisms as “elephant triggers” based on Lakoff’s book, **Do not Think of an Elephant**, where he suggests that mentioning an elephant – even if only to urge an audience *not* to think of an elephant – makes them immediately think of one (32). Lakoff shows that when advocates raise the frames or messages they intend to counter – even to criticize or undermine them – they may unintentionally reinforce them, or even suggest counter arguments their audience had not considered. Such “elephant triggers” can have implications for framing and effective communication by potentially creating barriers to conveying advocates’ own messages.

Moreover, the frequent use of advertising images that attractively depict SSBs or company brand names may have unintentionally reinforced positive associations with SSBs and the brands. Although these images are intended to depict the “target” of SSB tax policies, news sources and public health advocates inadvertently promoted SSBs and the beverage companies that manufacture them. Although there is limited guidance on the design of images for public health communications, research suggests that advocates should carefully consider how well their pictures align with text and how images can be used to strengthen key campaign messages (14). Advocates could consider using images that more clearly convey the goals of SSB taxes – for example, to hold the beverage industry accountable for health harms – to provide audiences with alternative frames in a media environment already saturated with beverage industry advertising and imagery.

The potential for “elephant triggers” may have worsened in recent years. Since Twitter was sold and rebranded to X in 2023, several changes were made to the platform. One notable change is how news stories appear: now posts that link to third-party news stories automatically load the article’s lead image and remove headlines, which may reduce necessary context (33). Given the volume of posts from news sources and the frequent depictions of SSB products and brands illustrating their stories as we found in our analysis, the potential effects of these changes to how news-related content is presented may inadvertently promote SSBs without providing critical context about SSB tax policies that may appear in headlines.

Our findings highlight the critical importance of images for advocates planning and disseminating SSB tax campaigns, including social media campaigns, and highlight the need to carefully select images that align with their overall goals and values without images of SSB products. Our analysis did, indeed, find a few posts that avoided repeating opposition frames, and instead shared images that portrayed how communities could thrive were SSB taxes in effect. These posts illustrate that advocates *can* convey values of health, equity, and community without raising beverage industry frames.

Our research focuses only on the content of Tweets about SSB taxes and not on their impact. For example, we could not directly determine whether the use of industry frames had any counter effects to pro-tax frames. Further, we did not analyze the number of views or “reach” per post, which could have provided insights into what types of arguments and images were more successful at reaching more audiences.

Our research is also limited because the availability of data was contingent on the Twitter application programming interface (API) and, due to the lack of data available prior to 2015, our sample may have underrepresented posts about the Berkeley SSB tax. In addition, retrospective nature of this study may not reflect real-time activity as some posts or accounts may have been deleted before we collected the data. Since we were unable to rely on Keyhole to collect posts by geographic region, we may have excluded California-based posts if the profile did not provide a location. The exclusion of posts from users who self-identified living outside of California may also have limited our analysis. Finally, we did not include “grocery tax” or opposition campaign names like “Yes to Affordable Groceries” in our search terms, which may have contributed to the low volume of posts more overtly against SSB taxes.

Despite these limitations, our research also suggests interesting avenues for future study. Although we found that news sources were frequently posted in our analysis, traditional print news outlets have declined in their circulation and reach (34). On the other hand, social media platforms have become increasingly popular sites for gathering news. For example, one-third of American adults recently reported that they regularly get their news from the short-form video platform TikTok (35). Further research on SSB tax campaigns on other social media platforms will also be important as such social media platforms evolve in their content delivery, accessibility, and popularity. However, since X has implemented paid subscriptions for access to its data (API), restricting what was previously free and unlimited access to Twitter data for academic research (36), researchers may face barriers to conducting similar studies in the future.

Our findings show that supporters effectively shared pro-tax messages in Tweets about SSB taxes, though sometimes overlooked how well their images aligned with those messages. These findings highlight an important opportunity for advocates planning campaigns for SSB tax policies to reduce consumption of SSBs. In the face of competing frames, particularly from the well-resourced beverage industry, public health advocates should continue to counter opposition with clear messages that explain the importance and value of the policy, supported by images that reinforce the messages and outcomes they want to achieve.

## Data availability statement

The raw data supporting the conclusions of this article will be made available by the authors, without undue reservation.

## Author contributions

KG: Data curation, Formal analysis, Investigation, Methodology, Resources, Validation, Visualization, Writing – original draft, Writing – review & editing. PM: Conceptualization, Data curation, Formal analysis, Investigation, Methodology, Project administration, Resources, Supervision, Validation, Visualization, Writing – original draft, Writing – review & editing. SP-S: Data curation, Formal analysis, Investigation, Methodology, Resources, Software, Validation, Visualization, Writing – original draft, Writing – review & editing. LD: Conceptualization, Project administration, Supervision, Writing – original draft, Writing – review & editing. KM: Conceptualization, Funding acquisition, Supervision, Writing – original draft, Writing – review & editing. DS: Conceptualization, Funding acquisition, Supervision, Writing – original draft, Writing – review & editing.
